# Panallergens and their impact on the allergic patient

**DOI:** 10.1186/1710-1492-6-1

**Published:** 2010-01-18

**Authors:** Michael Hauser, Anargyros Roulias, Fátima Ferreira, Matthias Egger

**Affiliations:** 1 Christian Doppler Laboratory for Allergy Diagnosis and Therapy, Department of Molecular Biology, University of Salzburg, Hellbrunnerstrasse 34, A-5020 Salzburg, Austria

## Abstract

The panallergen concept encompasses families of related proteins, which are involved in general vital processes and thus, widely distributed throughout nature. Plant panallergens share highly conserved sequence regions, structure, and function. They are responsible for many IgE cross-reactions even between unrelated pollen and plant food allergen sources. Although usually considered as minor allergens, sensitization to panallergens might be problematic as it bears the risk of developing multiple sensitizations. Clinical manifestations seem to be tightly connected with geographical and exposure factors. Future population- and disease-based screenings should provide new insights on panallergens and their contribution to disease manifestations. Such information requires molecule-based diagnostics and will be valuable for developing patient-tailored prophylactic and therapeutic approaches. In this article, we focus on profilins, non-specific lipid transfer proteins, polcalcins, and Bet v 1-related proteins and discuss possible consequences of panallergen sensitization for the allergic patient. Based on their pattern of IgE cross-reactivity, which is reflected by their distribution in the plant kingdom, we propose a novel classification of panallergens into ubiquitously spread "real panallergens" (e.g. profilins) and widespread "eurallergens" (e.g. polcalcins). "Stenallergens" display more limited distribution and cross-reactivity patterns, and "monallergens" are restricted to a single allergen source.

## Introduction

So far, from more than 200,000 known plant species, about 50 are registered in the official allergen list of the International Union of Immunological Societies (IUIS) Allergen Nomenclature Subcommittee http://www.allergen.org as capable of inducing pollen allergy in susceptible individuals [1]. Pollinosis-associated plants are characterized by production of high amounts of mostly anemophilous pollen and can be grouped as (i) trees (*Fagales, Pinales, Rosales, Arecales, Scrophulariales, Junglandales, Salicales*, and *Myrtales*), (ii) grasses (*Bambusioideae, Arundinoideae, Chloridoideae, Panicoideae*, and *Poideae*), and (iii) weeds (*Asteraceae *and *Chenopodiaceae*, and *Urticaceae*). The flowering seasons of allergenic plants spans the whole year, starting from early spring (trees), going over summer (grasses) and to late autumn (weeds). Allergenic pollen is a complex mixture of several molecules including major and minor allergens. Major allergens represent components to which the majority of patients (by definition >50%) reacting to a given allergen source is sensitized, whereas minor allergens are recognized by a limited number of patients. In many cases major allergens serve as marker allergens for sensitization to certain kinds of plants, e.g. Bet v 1 for birch, Cry j 1 and Cry j 2 for *Coniferales *allergies, Ole e 1 for *Oleaceae *[1], etc.

The number of allergic individuals that appears to be mono-sensitized to a single allergenic plant is very limited. In fact, the majority of patients seems to display adverse reactions upon contact to multiple allergen sources. According to the botanical classification, this might be simply attributed to poly-sensitization to different allergenic plants [2]. Another explanation for this phenomenon is the concept of IgE cross-reactivity in which IgE antibodies originally raised against a given allergen can bind homologous molecules originating from a different allergen source. For example, homologous molecules of the birch pollen major allergen Bet v 1 can be found in pollen of evolutionary related *Fagales *trees (e.g. alder Aln g 1, hornbeam Car b 1, chestnut Cas s 1, hazel Cor a 1, beech Fag s 1, oak Que a 1) and *Apiaceae *vegetables (e.g. celery Api g 1, carrot Dau c 1). However, botanical classification based on the allergenic source cannot explain the phenomenon of IgE cross-reactivity between evolutionary unrelated plant species. In this context, it should be mentioned that Bet v 1 homologues have also been identified in *Rosaceae *fruits (e.g. apple Mal d 1, cherry Pru av 1, apricot Pru ar 1, pear Pyr c 1), as well as in legumes, nuts, and seeds (e.g. hazelnut Cor a 1, soybean Gly m 4, peanut Ara h 8) [3-5]. This problem can only be properly analyzed from the allergen perspective, thus there is need to shift from a botanical to a molecular classification. Following this line, allergenic molecules have been integrated into families according to structural similarities. So far, 28 major groups of cross-reactive proteins have been identified, *i.e. *6 groups of pathogenesis-related (PR) proteins, 11 groups of various enzymes (e.g. proteases, glycolytic enzymes, *etc.*), and others, such as transport proteins, protease inhibitors, and regulatory as well as structural proteins [4].

Molecular classification offers the possibility to explain allergy to multiple pollen and pollen-related food allergies. As mentioned above, PR proteins of Bet v 1-related molecules can be found in the pollen of *Fagales *trees, and in foods belonging to various botanical families being responsible for adverse reactions upon contact to both pollen and food allergen sources. Thus, based on IgE-recognition, the family of Bet v 1-related proteins can be defined as a cross-reactivity cluster (Table 1). However, among certain homologous allergens little or no cross-reactivity has been observed. Therefore, the molecular definition of cross-reactivity clusters cannot solely rely on sequence homology but requires experimental studies [1].

**Table 1:** Members of panallergen families and of the Bet v 1 cluster

panallergen family	plant allergen source							
	pollen			food				product
	
	trees	grasses	weeds	fruits	vegetables	legumes	nuts/seeds	latex
**profilins**	Bet v 2	Cyn d 12	Amb a 8	Act d 9	Api g 4	Gly m 3	Ara h 5	Hev b 8
	Car b 2	Lol p 12	Art v 4	Ana c 1	Cap a 2		Cor a 2	
	Cor a 2	Ory s 12	Che a 2	Cit s 2	Dau c 4		Pru du 4	
	Fra e 2	Phl p 12	Hel a 2	Cuc m 2	Lyc e 1			
	Ole e 2	Poa p 12	Mer a 1	Fra a 4				
	Pho d 2	Zea m 12	Par j 3	Lit c 1				
				Mal d 4				
				Mus xp 1				
				Pru du 4				
				Pru av 4				
				Pru p 4				
				Pyr c 4				

**polcalcins**	Aln g 4	Cyn d 7	Amb a 9					
	Bet v 3	Phl p 7	Amb a 10					
	Bet v 4		Art v 5					
	Fra e 3		Che a 3					
	Jun o 4							
	Ole e 3							
	Ole e 8							
	Syr v 3							

**nsLTPs**	Ole e 7		Amb a 6	Act c 10	Api g 2		Ara h 9	Hev b 12
	Pla a 3		Art v 3	Act d 10	Aspa o 1		Cas s 8	
			Hel a 3	Cas s 8	Bra o 3		Cor a 8	
			Par j 1	Cit l 3	Lac s 1		Jug r 3	
			Par j 2	Cit s 3	Lyc e 3			
			Par o 1	Fra a 3	Zea m 14			
				Mal d 3				
				Pru ar 3				
				Pru av 3				
				Pru d 3				
				Pru du 3				
				Pru p 3				
				Pyr c 3				
				Vit v 1				

**Bet v 1 cluster**	Aln g 1			Act c 8	Api g 1	Gly m 4	Ara h 8	
	Bet v 1			Act d 8	Dau c 1	Vig r 1	Cor a 1.04	
	Car b 1			Ara h 8				
	Cas s 1			Mal d 1				
	Cor a 1			Pru ar 1				
	Fag s 1			Pru av 1				
	Que a 1			Pru p 1				
				Pyr c 1				

Currently known plant profilins, polcalcins, nsLTPs, and members of the Bet v 1 family of allergens http://www.allergen.org.

## Panallergens

**Figure 1:**
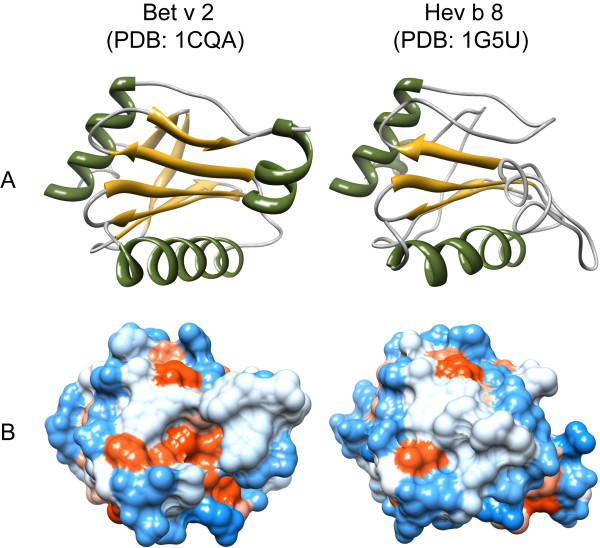
**Three-dimensional structures of allergenic profilins**. Secondary structure elements (A) are displayed in green (α-helices) and yellow (β-sheets). The distribution of hydrophilic (blue) and hydrophobic (red) amino acids over the molecular surface is depicted in B. All models were obtained from the Protein Structure Database http://www.pdb.org/pdb/home/home.do and visualized with chimera http://www.cgl.ucsf.edu/chimera/

**Figure 2:**
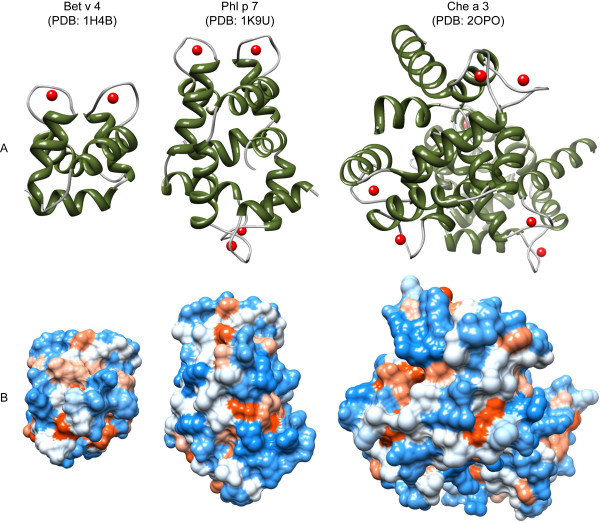
**Three-dimensional structures of allergenic polcalcins**. Monomeric birch Bet v 4, dimeric timothy grass Phl p 7, and tetrameric goosefoot Che a 3 represent 2EF-polcalcins from tree, grass, and weed pollen. Molecules are depicted in their "holo"-conformation with bound calcium ions illustrated as red balls. Secondary structure elements (A) are shown in green (α-helices) and yellow (β-sheets). The distribution of hydrophilic (blue) and hydrophobic (red) amino acids over the molecular surface is depicted in B. All models were obtained from the Protein Structure Database http://www.pdb.org/pdb/home/home.do and visualized with chimera http://www.cgl.ucsf.edu/chimera/

**Figure 3:**
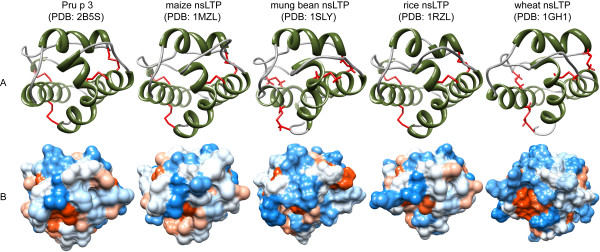
**Three-dimensional structures of nsLTPs**. NsLTPs share a common fold that is composed of 4 α-helices (highlighted in green) and stabilized by 4 disulfide bonds (shown in red) to form a central tunnel for ligand interaction (A). The distribution of hydrophilic (blue) and hydrophobic (red) amino acids over the molecular surface is depicted in B. All models were obtained from the Protein Structure Database http://www.pdb.org/pdb/home/home.do and visualized with chimera http://www.cgl.ucsf.edu/chimera/

**Figure 4:**
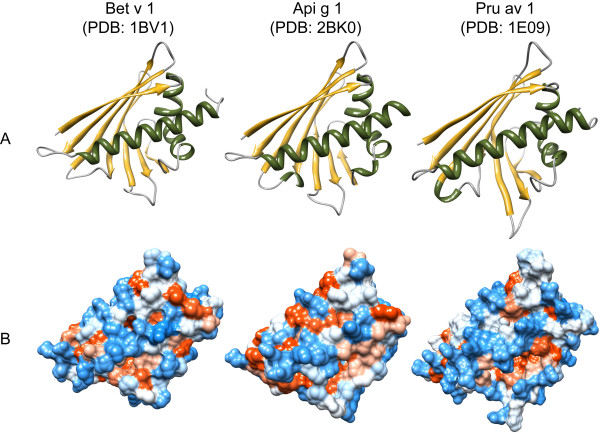
**Three-dimensional structures of birch pollen Bet v 1 and homologous food allergens**. Structures reveal a typical alpha/beta fold that is responsible for IgE cross-reactivity among related and unrelated species. Secondary structure elements (A) are displayed in green (α-helices), yellow (β-sheets), and grey (loops and turns). The distribution of hydrophilic (blue) and hydrophobic (red) amino acids over the molecular surface is depicted in B. All models were obtained from the Protein Structure Database http://www.pdb.org/pdb/home/home.do and visualized with chimera http://www.cgl.ucsf.edu/chimera/

In addition to major allergens, also minor allergens have been shown to be responsible for cross-recognition of unrelated plant species. Many minor allergens are involved in general vital functions and can therefore be widely found from plants to men. This gives rise to the so called "panallergen" concept, with the Greek prefix "pan" meaning "all", emphasizing the ubiquitous distribution of some minor allergenic molecules throughout nature. Although originating from unrelated organisms, such functionally related molecules share highly conserved sequence regions and three-dimensional structures and hence, can fulfill the requirements for IgE cross-recognition. Known panallergens presently comprise only a few protein families, including profilins, polcalcins, and non-specific lipid transfer proteins (nsLTP). Multiple allergies to both pollen and food allergen sources seem to be determined by sensitization to such ubiquitously spread allergens [6]. In fact, polysensitization to different allergen sources is more frequently observed in patients displaying profilin-specific IgE antibodies [7,8]. These findings can be explained by extensive IgE cross-reactivity between panallergens from different sources [9], but also by cross-allergenicity underlying the T cell response to conserved regions of panallergens [10]. This circumstance is highly relevant in the management of patients with multiple allergies and possibly for the development of multiple allergies [2]. Initial exposure to panallergens may subsequently drive the allergic immune response towards major allergens through a mechanism called intramolecular epitope spreading [11]. In the present article we focus on the panallergenic protein families of profilins, polcalcins, and nsLTPs and their clinical relevance for the allergic patient. Individual members of panallergen protein families are given in Table 1 and three-dimensional structures are illustrated in Figure 1, 2, 3 and 4. Panallergens that have been convincingly demonstrated to be clinically relevant in ragweed, timothy grass, and birch pollinosis-associated food allergies are listed in Table 2[3,5,12,13].

**Table 2:** Food allergies associated with pollinosis to common allergenic plants in Canada

Associated food allergen sources	Common pollen allergen sources in Canada		
	
	Ragweed	Timothy grass	Birch
**Fruits**	**banana **Mus xp 1: profilin [7]		**apple **Mal d 1 PR-10 [73-75]
	**melon **Cuc m 2: profilin [7]		**cherry **Pru av 1: PR-10 [26,73,75]
		**orange **Cit s 2: profilin [7]	
			**kiwi**
			**peach **Pru p 1: PR-10 [73]
			**pear**
			**plum**
	**watermelon ** profilin [7]		
**Vegetables**	**cucumber**		**carrot **Dau c 1: PR-10 [75] Dau c 4: profilin [73]
	**zucchini**		**celery **Api g 1: PR-10 [75] Api g 4: profilin [73]
		**potato**	
		**tomato **Lyc e 1: profilin [7]	
**Legumes**			**soybean **Gly m 4: PR-10 [76]
**Nuts/Seeds**			**almond**
			**hazelnut **Cor a 1: PR-10 [75]
			**other nuts**
		**peanut **Ara h 8 [5]	

The individual profilins and members of the Bet v 1 allergen family (PR-10 proteins) listed in the table have been convincingly demonstrated to be of clinical relevance in ragweed, timothy grass, and birch pollinosis-associated food allergies [3,5,12,13] by *in vivo *(SPT) or *in vitro *(mediator release) assays [5,7,26,74-77]. A picture is now emerging in which profilins seem to be responsible for pollinosis-associated allergy to non-*Rosaceae *fruits (ragweed Amb a 8 and timothy grass Phl p 12). PR-10 proteins (Bet v 1) and to a minor extent profilins (Bet v 2) appear to be involved in food incompatibilities associated with birch pollinosis. Sensitization to nsLTPs seems to be linked to pollinosis-independent class I food allergies [68,69]. Expression of polcalcins is restricted to pollen tissue and therefore, they do not play a role in pollen-associated food allergies [34].

## Profilins

Profilins represent a family of small (12 to 15 kDa), highly conserved molecules sharing sequence identities of more then 75% even between members of distantly related organisms. This sequence conservation is reflected by highly similar structures and biologic function [4]. Profilins can be found in all eukaryotic cells and are involved in processes related to cell motility via regulation of microfilament polymerization upon binding to actin [14]. In plant cells, profilins play a role in cytokinesis, cytoplasmatic streaming, cell elongation as well as growth of pollen tubes and root hairs [15-17]. Besides actin, a plethora of profilin ligands have been described, e.g. phosphoinositides and poly-L-proline stretches, providing evidence for profilin involvement in other cellular processes like membrane trafficking and organization as well as signaling pathways [18]. Being a component of many essential cellular processes, profilins are ubiquitously spread and can therefore be viewed as panallergens that are responsible for many cross-reactions between inhalant and nutritive allergen sources [14,19]. In accordance, allergenic profilins were identified in pollen of trees, grasses, and weeds, in plant-derived foods, as well as in latex (Table 1). IgE cross-reactivity results from the common three-dimensional profilin fold composed of two α-helices and a five-stranded anti-parallel β-sheet, as described for the class of α-β proteins [4] (Figure 1). Due to this conserved structure, profilin-specific IgE may cross-react with homologues from virtually every plant source. Therefore, profilin sensitization is a risk factor for allergic reactions to multiple pollen and food allergen sources [20].

The first allergenic profilin was described in birch pollen and was designated Bet v 2 [19]. Shortly after the identification of Bet v 2, IgE cross-reactive profilins were found in pollen of grasses and weeds [14]. Cross-reactivity between the weed pollen profilins Art v 4 (mugwort) and Amb a 8 (ragweed) has been convincingly demonstrated, although the same study delivered evidence that allergic individuals with positive skin prick tests to ragweed and mugwort pollen were co-sensitized [21]. Furthermore, hazelnut Cor a 2 and *Rosaceae *profilins (strawberry Fra a 4, apple Mal d 1, cherry Pru av 4, almond Pru du 4, peach Pru p 4, and pear Pyr c 4) are considered to cross-react with grass and birch profilins [22]. Interestingly, most of the plant food-derived profilins characterized so far have been shown to be involved in pollen food cross-reactive syndromes. As profilins are sensitive to heat denaturation and gastric digestion, they cannot cause sensitization via the gastrointestinal tract. In fact, consumption of raw foods by profilin-sensitized patients leads to reactions that are usually restricted to the oral cavity [23,24]. Such properties are typical for class II food allergens. In contrast to non-pollen-related class I food allergy that mainly affects young children, class II food incompatibility is frequently observed in adults as a consequence of sensitization to aeroallergens [25]. In this context, allergic cross-reactions between ragweed, melon, and banana seems to be mediated by profilins, *i.e. *Amb a 8, Cuc m 2, and Mus xp 1, respectively. Moreover, allergic reactions to celery and carrot profilins, Api g 4 and Dau c 4, were observed in patients with concomitant birch or mugwort pollinosis (Table 2). Interestingly, Api g 4 was shown to display partial heat resistance, and consequently might also elicit symptoms after heat treatment [26,27]. In addition, profilins have been described to mediate cross-reactions between pollen and exotic fruit, like lychee Lit c 1 and pineapple Ana c 1.

Profilin sensitization varies between 5 to 40% among allergic individuals. This variability was addressed by previous studies suggesting that the allergenic source, levels of exposure, and geographical factors influence profilin sensitization. For example, in 1997 Elfman *et al. *[28] reported different profiles for specific IgE to Bet v 1 and Bet v 2 in birch pollen-allergic patients. As revealed by immunoblot analyses, 100% of the sera derived from Northern European subjects displayed reactivity with Bet v 1, but only 5 to 7% reacted with birch profilin. In contrast, 20 to 38% of a Central/Southern European group was positive for Bet v 2. Similarly, it was shown that among weed pollen-allergic patients sensitization to mugwort and ragweed profilins (Art v 4 and Amb a 8, respectively), was much lower in Italians (20%) when compared to the Austrian (45 to 50%) population [29].

The clinical relevance of profilin sensitization is still a matter of debate. One study examining the cross-reactivity patterns of IgE antibodies from birch pollen-allergic patients with concomitant food allergy [30] showed that in contrast to Bet v 1-specific IgE, antibodies directed against birch profilin have a broad cross-reactivity spectrum. In fact, Bet v 2 sensitization was associated with positive RAST (radio allergosorbent test) to all investigated plant-derived foods except apple, peach, and melon. However, their clinical relevance was low or even absent. By contrast, Bet v 1-specific IgE frequently gave rise to clinically relevant cross-reactivities. In contrast, Asero *et al. *[7] showed that more than half of investigated profilin-sensitized patients display clinically relevant cross-sensitization to plant-derived foods leading to the conclusion that profilins can be considered clinically relevant food allergens (Table 2). Furthermore, the authors suggested that allergy to melon, watermelon, tomato, banana, pineapple, and orange can be considered as markers of profilin hypersensitivity in Mediterranean countries [20].

Olive profilin Ole e 2 has been reported to cross-react with grass profilins [31], and Ole e 2-specific IgE antibodies were detected in 95% of olive pollen-allergic patients with concomitant oral allergy syndrome to peach, pear, melon, kiwi, and nuts [32]. Furthermore, a statistically significant association between sensitization to both Ole e 2 and the glucanase homologue Ole e 10 with the development of bronchial asthma has been reported [33]. These studies emphasize the importance of identifying the responsible allergens as they might have an impact on the clinical features of allergic reactions to fruits and vegetables.

Taken together, patients displaying profilin-specific IgE antibodies are either sensitized or at risk of developing multiple pollen sensitization and pollen-associated food allergy. Thus, despite the fact that many profilin-sensitized patients do not exhibit symptoms, careful patient monitoring and a clear distinction between cross-reactivity and genuine sensitization seem advisable for the reasons stated above.

## Polcalcins

Polcalcins are a group of allergens belonging to the family of calcium-binding proteins (CBP) sharing common domains termed EF-hands (helix-loop-helix motifs). Besides polcalcins, the EF-hand superfamily of proteins includes a panel of allergenic proteins like parvalbumins from fish and amphibian food, as well as cockroach Bla g 6, mite Der f 17, cattle Bos d 3, and man Hom s 4. Polcalcins constitute the majority of allergenic CBPs, and their expression seems to be restricted to pollen (Table 1). According to the number of calcium-binding EF-hand motifs, at least three types of polcalcins have been described in pollen, *i.e. *those displaying two (Aln g 4, Amb a 9, Art v 5, Bet v 4, Che a 3, Cyn d 7, Fra e 3, Ole e 3, Phl p 7, and Syr v 3), three (Amb a 10 and Bet v 3), and four (Jun o 4, and Ole e 8) calcium-binding domains. The three-dimensional structure of polcalcins is characterized by α-helices displaying a typical all α protein fold. The monomer, displaying a molecular weight of 8 to 9 kDa, shows the typical polcalcin structural domain. For example, monomeric Bet v 4 from birch is composed of two symmetrically arranged EF-hands bringing the two bound calcium ions into spatial proximity. Dimeric timothy grass Phl p 7 contains two of these basic structural domains; four of these domains were observed in the tetrameric goosefoot Che a 3 (Figure 2). The biologic function of polcalcins is still unclear. However, due to their pollen-specific localization and their ability to bind calcium, it has been proposed that polcalcins function in the control of intracellular calcium levels during pollen germination [34]. Interestingly, the calcium-binding property of polcalcins affects both the molecule's IgE-reactivity and thermostability. Calcium association induces conformational changes in the three-dimensional structure and two conformational states of CBPs can be distinguished, *i.e. *the closed calcium-free "apo", and the open calcium-associated "holo" forms. Several studies demonstrated that the apo-forms are less stable to thermal denaturation and display decreased IgE-reactivity when compared to their calcium-bound counterparts [35-40]. Moreover, a comparative study between allergens with two, three, and four EF-hand domains revealed that timothy grass Phl p 7 is the most cross-reactive polcalcin. It has therefore been suggested that Phl p 7 could serve as marker molecule for the identification of multiple pollen sensitizations [41]. Enhanced IgE binding of Phl p 7 was tentatively attributed to its capacity to form dimers [42,43]. However, studies comparing monomeric Bet v 4, dimeric Phl p 7, and tetrameric Che a 3 are lacking.

Taken together, polcalcins are highly cross-reactive calcium-binding allergens that are specifically expressed in pollen tissues. For this reason, sensitization to polcalcins is not associated with allergy to plant-derived foods. Approximately 10% of pollinosis patients react with polcalcins from various trees, grasses, and weeds [21,42]. Recent data indicate that the clinical relevance of polcalcin sensitization is linked to geographical factors and level of exposure to different allergenic sources. It has been shown that among weed pollen-allergic patients, reactivity to the polcalcins Art v 5, Amb a 9, and Amb a 10 from mugwort and ragweed, respectively, was much higher in Italians (21 to 28%) when compared to an Austrian (10%) group [29], indicating that positive IgE results do not exclusively reflect cross-reactivity but also indicate sensitization to mugwort or ragweed in these populations. Hence, careful monitoring of polcalcin-sensitized patients should be performed as these individuals are at risk of developing multiple pollen sensitizations.

## Non-specific lipid transfer proteins

Non-specific lipid transfer proteins (nsLTPs), originally named after their ability to bind and enhance the transfer of a multitude of different types of lipid molecules between membranes *in vitro*, constitute a family of 7 kDa (nsLTP 2 subfamily) or 9 kDa (nsLTP 1 subfamily) proteins that are widely distributed throughout the plant kingdom. However, a role in plant intracellular trafficking of membrane lipids *in vivo *seems unlikely. A possible role of nsLTPs in the transport of cutin and suberin monomers to the outer layer of plant organs has been reported [44]. This is consistent with data showing that nsLTPs are located in the peel of fruits rather than in the pulp [45,46]. Potential involvement of nsLTPs in plant growth and development, including embryogenesis, germination, and pollen-pistil interaction has also been suggested [47].

nsLTPs belong to the class of pathogenesis-related (PR) proteins [48], and are thought to play a role in plant defense due to their antifungal and antibacterial activities. PR-proteins comprise 14 unrelated protein families, which by definition are induced upon environmental stress, pathogen infection, and antibiotic stimuli. nsLTPs represent the PR-14 family, which is characterized by a common fold of four α-helices stabilized by four disulfide bonds that form a central hydrophobic tunnel interacting with lipid molecules (Figure 3). Interestingly, another PR-protein family, *i.e. *the PR-10 family of Bet v 1-related proteins [4], has been also shown to represent cross-reactive plant allergens.

Allergenic nsLTPs have been identified in the pollen of trees and weeds, in plant food allergen sources, and in latex. Curiously, nsLTPs have not been identified yet in grass pollen (Table 1). The allergenic potential of nsLTPs is influenced by several factors *i.e. *localization and stability to proteolytic and thermal denaturation. It has been demonstrated that nsLTPs are stable molecules predominantly present in the peel of fruits [45,46], which might explain why some LTP-sensitized individuals can more easily tolerate fruits after peeling. This aspect was recently addressed by Borges *et al. *[49], investigating nsLTP localization in different *Rosaceae *fruits. The authors showed that except for plum and apricot, nsLTPs are indeed concentrated in the skin. As revealed by immunolocalization, nsLTPs are primary located in the cytosol and are subsequently excreted to accumulate in the cell wall. The hairy peel of peach is particularly rich in nsLTPs. In this context, it is notable that anaphylactic responses have been reported in Spanish patients just after skin contact with peach [50]. Furthermore, differences in the content of nsLTPs among various commercially available kinds of apple have been observed. This knowledge is especially important for weakly sensitized *Rosaceae*-allergic patients as they can reduce the risk of severe allergic reactions by avoiding certain kind of fruits or by consuming peeled-off fruits. This is also important concerning sensitization because nsLTPs can act as true food allergens with the capacity to induce severe symptoms by surviving food processing and the harsh environment of the gastrointestinal tract [51] due to their high resistance to heat and proteolysis.

In the Mediterranean area, allergy to *Rosaceae *fruits is associated with sensitization to nsLTPs, which are regarded as major allergens in those countries. By contrast, sensitization to nsLTPs is rarely observed in central and northern Europe, where allergy to *Rosaceae *fruit is more often associated with Bet v 1 [32,47,52,53] (Table 2). It has been speculated that these geographical differences could be explained by differences in food consumption and pollen exposure, e.g. birch pollen in Northern and Central Europe, and pollen of olive, plane tree, and pellitory in Mediterranean countries. However, the question whether pollen or food nsLTPs act as primary sensitizers still remains unanswered [51].

Recent studies on cross-reactivity of nsLTPs showed that most *Rosaceae*-allergic and nsLTP mono-sensitized patients experience adverse reaction after ingestion of botanically unrelated plant-derived foods as well. The most frequently reported causes of allergic symptoms were nuts (hazelnut, walnut, and peanut). By contrast, carrot, potato, banana, and melon seemed to be safe for LTP-allergic patients as indicated by lack of IgE reactivity, negative case history and skin prick tests (SPT), and confirmation by open oral challenge [54,55]. Besides allergy to *Rosaceae *fruits, nsLTPs have also been reported to play a key role in chestnut allergy. Adverse reactions to chestnuts are usually associated with allergy to latex within the latex-fruit syndrome that is mainly caused by class I chitinases and latex hevein cross-reactive allergens. In this respect, chestnut nsLTP (Cas s 8) has been proposed as a marker allergen for chestnut-allergic patients without concomitant latex hypersensitivity [56].

Taken together, nsLTPs are major cross-reactive allergens identified in the majority of plant-derived foods as well as in pollen from diverse plants. Sensitization to nsLTPs is characterized by geographical differences, presumably several routes of sensitization, and often associated with severe symptoms of food allergy. Patients displaying *Rosaceae *nsLTP-specific IgE antibodies often tolerate peeled-off fruits, and certain foods, such as carrots, potatoes, bananas, and melon, but are at risk of developing allergic reactions upon ingestion of nuts. This knowledge is important for a better management of allergy to nsLTPs.

## Diagnostic and therapeutic aspects of panallergens

Currently, allergen extracts are used for both allergy diagnosis and immunotherapy, which presently is the only curative approach towards the treatment of allergy. However, currently used allergenic extracts contain mixtures of allergens, non-allergenic and/or toxic proteins, bearing the risk of IgE-mediated side effects and sensitization to new allergens. Moreover, standardization of allergenic extracts still relies on the usage of company-specific units, rendering impossible comparison between commercial allergenic products from different manufacturers. In addition, relevant allergens for a given patient might be underrepresented or even missing in the extract used for diagnosis or therapy [57]. This might be especially true for minor allergens, such as panallergens. However, sensitization to panallergens might worsen the prognosis of allergy due to extensive IgE cross-reactivity towards evolutionary related and unrelated allergen sources or, as in the case of nsLTPs, increase severity of atopic disease [58]. For example, olive pollen exposure levels seem to influence patient's sensitization profiles. Patients from areas with low pollen counts are mainly sensitized to the major allergen Ole e 1. However, exposure to high levels of olive pollen dramatically increases the frequency and levels of IgE antibodies specific for minor allergens, as well as the severity of allergic disease. Standardization of allergenic extracts is usually based on the concentration of the main IgE-binding molecule. Therefore, such extracts might not be adequate for diagnosing and treating patients reacting to minor allergens [59]. The problems discussed above could be solved by molecule-based diagnostics and custom-tailored immunotherapy using a panel of naturally purified or recombinantly produced allergens [60]. As reactions to pollen originating from multiple sources are frequently due to sensitization to conserved allergens (panallergens) rather than to genuine sensitization due to exposure to pollen from various species, diagnosis based on allergenic molecules seems to be especially important for multiple-sensitized patients [6]. In this context, it has even been shown that only a single plant profilin may be used for diagnosis of patients suffering from multiple pollen sensitization and/or pollen-associated food allergy. Indeed, there is increasing evidence that well-defined marker allergens available as recombinant proteins may be used for helping the decision-making process in diagnosis and for monitoring currently available forms of specific immunotherapy [61-63].

## Discussion

Panallergens, commonly classified as minor allergens, are ubiquitous proteins responsible for IgE cross-reactivity to a wide variety of related and unrelated allergenic sources. Usually, IgE cross-reactivity is seen from the allergen-perspective, meaning cross-reactivity is a consequence of structural similarity between homologous proteins, which is translated into conserved sequence regions, three-dimensional folding, and function. However, it has been shown that antibodies also can contribute to cross-reactivity by means of conformational diversity [64]. In an interesting study, James *et al. *demonstrated that a single antibody molecule could adopt different paratope conformations, thereby binding to unrelated antigens. Such promiscuous antibody isomers can effectively increase the size of the antibody repertoire and may also lead to cross-reactivity and disease. Moreover, as humoral antibody responses require T cell assistance, cross-reactivity can be also discussed at the cellular level. Although our current knowledge on this topic in association with allergic disease is quite limited, T cell cross-allergenicity might be a crucial issue for better understanding of polysensitization and the role of panallergens. For example, Burastero *et al. *[2] recently reported that initial exposure of T cells to conserved pollen panallergens can extend the immune response towards other allergenic components leading to novel sensitization. T cell cross-reactivity has also been investigated in pollen-related food allergy. Cross-reactive T cell epitopes of Bet v 1-related food allergens, which where not destroyed by gastrointestinal digestion, stimulated Bet v 1-specific T cells *in vitro *despite the IgE non-reactivity of the food allergen. Similarly, cooked food allergens were unable to elicit IgE-mediated symptoms but caused T cell-mediated late phase reactions in birch pollen-allergic patients. Thus, T cell cross-reactivity might have implications for the pollen-specific immune response of allergic individuals [65].

Taken together, understanding of immunologic cross-reactivity is essential to advance our knowledge about allergy. Additionally, this knowledge might help in the development of intelligent tools for the prediction of allergenicity of novel proteins or foods [66] to which individuals previously have not been exposed. In fact, profilins, nsLTPs, and a Bet v 1 homologue were identified in vegetable varieties that were recently introduced to the European market [67].

In contrast to polcalcins that only can be found in pollen, profilins and nsLTPs are generally regarded as panallergens being involved in cross-reactions between pollen and food allergen sources. The question is now emerging, if members of the Bet v 1 family of allergens could also be considered as panallergens? Panallergens, usually classified as minor allergens, are defined as homologous molecules that originate from a multitude of organisms and cause IgE cross-reactivity between evolutionary unrelated species. Bet v 1 homologues represent major allergens in pollen of *Fagales *but can also be found in many allergenic foods belonging to the botanical orders of *Apiales*, *Ericales*, *Fagales*, and *Rosales *(Table 1), and their similar structures (Figure 4) give rise to many birch-pollinosis associated food allergies (Table 2). By definition, next to profilins, polcalcins, and nsLTPs, Bet v 1 homologues might therefore be integrated as a forth group of panallergenic proteins. If so, the panallergen concept should be redefined. Among panallergen families, only profilins seem to be distributed ubiquitously throughout the plant kingdom. As they are responsible for allergic reactions against a multitude of evolutionary unrelated pollen and nutritive allergen sources, profilins could be classified as "real panallergens". By contrast, the distribution of nsLTPs, PR-10 proteins, and in particular polcalcins seems to be more limited (Table 1), which is reflected by a more restricted pattern of IgE cross-reactivity. For example, Bet v 1- like proteins are involved in cross-reactions between *Fagales *pollen and plant-derived foods originating from only a small number of botanical families (*Rosaceae, Apiaceae, Actinidiaceae, and Fabaceae*) (Table 2). Occurring exclusively in pollen grains of plants, polcalcins are not involved in pollinosis-associated plant food allergies at all [34]. Although being expressed in a greater variety of plant tissues, sensitization to nsLTPs is rather linked to pollinosis-independent class I food allergy [68,69]. Such allergens would rather not deserve the designation panallergen but could be classified as "eurallergens" with the Greek prefix "eu" (from euros: width) emphasizing their wide but not ubiquitous distribution in the plant kingdom. Following this line, we suggest to designate widespread allergens displaying limited cross-reactive patterns as "stenallergens" (Greek: "stenos": tight), and "monallergens" (Greek "monos": single), those which are restricted a few or a single botanical family (Table 3).

**Table 3:** Classification of plant allergen families according to their patterns of distribution and IgE cross-reactivity

Classification	Plant allergen family	Clinically relevant IgE cross-reactivity between unrelated allergen sources	Distribution							
			Pollen			Plant food				Product
			T	G	W	F	V	N	L	LA
**Panallergens**	Profilins	yes [7]	**X**	**X**	**X**	**X**	**X**	**X**	**X**	**X**

**Eurallergens**	Polcalcins	yes [34]	**X**	**X**	**X**					
	nsLTPs	yes [78]	**X**		**X**	**X**	**X**		**X**	**X**
	PR-10 proteins	yes [75]	**X**			**X**	**X**	**X**	**X**	
	Hevein-like domain proteins	yes [79]	**X**			**X**	**X**	**X**		**X**
	β-1,3 glucanases	yes [80]	**X**			**X**	**X**			**X**
**Stenallergens**	Ole e 1-related proteins	no	**X**	**X**	**X**					
	Polygalacturonases	no	**X**	**X**						
	Pectate lyases	no	**X**		**X**					
	Cyclophilins	no	**X**		**X**					
	Thaumatin-like proteins	no	**X**			**X**	**X**			
	Plant invertases	no	**X**			**X**				
	Isoflavone reductases	no	**X**			**X**				
	PR-1 proteins	no		**X**	**X**					
	Expansins (N-terminal)	no		**X**		**X**				
	α-amylase/trypsin inhibitors	no		**X**				**X**		
	Cystatins	no			**X**	**X**				
	Pectin methylesterases	no			**X**	**X**				
	Patatins	no					**X**			**X**
	Barwin family proteins	no					**X**			**X**
	Cupins	no						**X**	**X**	
	Fe/Mn Superoxide dismutases	no						**X**		**X**
	Thioredoxins	no						**X**		**X**

**Monallergens**	8 domain proteins	-	**X**							
	Heat shock proteins (Hsp70)	-	**X**							
	Expansins (C-terminal)	-		**X**						
	Group 5/6 grass allergens	-			**X**					
	Berberine bridge enzymes	-		**X**						
	Protein kinases	-			**X**					
	Group 5 ragweed allergens	-		**X**						
	Papain-like cysteine proteases	-				**X**				
	60S acidic ribosomal proteins	-				**X**				
	Kunitz-type trypsin inhibitors	-					**X**			
	Glycoside hydrolase family 32 proteins	-					**X**			
	Cereal prolamins	-						**X**		
	2S albumins	-						**X**		
	Oleosins	-						**X**		
	Serpin serine protease inhibitors	-						**X**		
	α-amylases	-						**X**		
	Legume lectins	-							**X**	
	Rubber elongation factors	-								**X**
	SGNH-hydrolases	-								**X**

Due to their pattern of distribution and IgE cross-reactivity between unrelated species, plant allergens can be classified as (i) ubiquitously spread cross-reactive "panallergens" (Greek "pan": all), (ii) widespread cross-reactive "eurallergens" (Greek "euros": width), (iii) "stenallergens" (Greek: "stenos": tight), widespread allergen with limited cross-reaction, and (iv) "monallergens" (Greek "monos": single), which are restricted to a single allergen source. Detailed information on allergen families is available from the AllFam database http://www.meduniwien.ac.at/allergens/allfam/[81]. (Abbreviations: T, trees; G, grasses; W, weeds; F, fruits; V, vegetables; N, nuts and seeds; L, legumes; LA, latex)

It is worth mentioning, that despite profilins and eurallergens, extensive IgE cross-reactivity among unrelated species is also caused by cross-reactive carbohydrate determinants (CCD) of glycoproteins that are widely distributed across evolutionary lineages. Indeed, carbohydrate-specific antibodies are abundant in humans [70,71]. Moreover, it has been reported that more then 20% of allergic patients produce anti-glycan IgE antibodies that bind to glycoproteins in pollen, foods, and insect venoms. However, the clinical relevance of carbohydrate-specific IgE is still a matter of debate. Compiled evidence suggests that CCDs do not cause clinical symptoms in most, if not all, allergic individuals [72]. Hence, CCDs would represent a special case of panallergenic structures responsible for IgE cross-reactivity with limited clinical relevance.

A picture is now emerging in which panallergens seem to be important players in the clinical manifestation of allergic sensitization, e.g. association with bronchial asthma in *Oleaceae*-sensitized patients, which seems to be tightly connected with geographical and exposure factors. The availability of well-characterized recombinant panallergens has paved the way to numerous studies focused on their clinical relevance. Future investigations aiming at population- and disease-based screenings should provide new and important insights on panallergens and their contribution to disease manifestations among pre-disposed individuals. Such information will be valuable for developing patient-tailored prophylactic and therapeutic approaches.

## Abbreviations

Act c: *Actinidia chinensis *(gold kiwi); Act d: *Actinidia deliciosa *(green kiwi); Aln g: *Alnus glutinosa *(alder); Amb a: *Ambrosia artemisiifolia *(ragweed); Ana c: *Ananas comosus *(pineapple); Api g: *Apium graveoles *(celery); Ara h: *Arachis hypogaea *(peanut); Art v: *Artemisia vulgaris *(mugwort); Aspa o: *Asparagus officinalis *(asparagus); Bet v: *Betula verrucosa *(birch); Bra o: *Brassica oleracea *(cabbage); Cap a: *Capiscum annuum *(bell pepper); Car b: *Carpinus betulus *(hornbeam); Cas s: *Castanea sativa *(chestnut); CBP: calcium-binding protein; CCD: cross-reactive carbohydrate determinant; Che a: *Chenopodium album *(goosefoot); Cit l: *Citrus limon *(lemon); Cit s: *Citrus sinensis *(orange); Cor a: *Corylus avellana *(hazel/hazelnut); Cuc m: *Cucumis melo *(melon); Cyn d: *Cynodon dactylon *(Bermuda grass); Dau c: *Daucus carota *(carrot); EF-hand: helix-loop-helix motif; Fag s: *Fagus sylvatica *(beech); Fra a: *Fragaria ananassa *(strawberry); Fra e: *Fraxinus excelsior *(ash); Gly m: *Glycine max *(soybean); Hel a: *Helianthus annuus *(sunflower); Hev b: *Hevea brasiliensis *(latex); IgE: immunoglobulin E; IUIS: International Union of Immunological Societies; Jug r: *Juglans regia *(walnut); Jun o: *Juniperus oxycedrus *(juniper); kDa: kilo Dalton; Lac s: *Lactuca sativa *(lettuce); Lit c: *Litchi chinensis *(litchi); Lol p: *Lolium perenne *(Ryegrass); Lyc e: *Lycopersicum esculentum *(tomato); Mal d: *Malus domesticus *(apple); Mer a: *Mercurialis annua *(mercury); Mus xp: *Musa x paradisiaca *(banana); nsLTP: non-specific lipid transfer protein; Ole e: *Olea europaea *(olive); Ory s: *Oryza sativa *(rice); Par j: *Parietaria judaica *(pellitory of the wall); Par o: *Parietara officinalis *(pellitory); Phl p: *Phleum pratense *(timothy grass); Pho d: *Phoenix dactylifera *(palm); Pla a: *Platanus acerifolia *(plantain); Poa p: *Poa pratensis *(Kentucky Blue grass); PR: pathogenesis related; Pru ar: *Prunus armeniaca *(apricot); Pru av: *Prunus avium *(cherry); Pru d: *Prunus domestica *(plum); Pru du: *Prunus dulcis *(almond); Pru p: *Prunus persica *(peach); Pyr c: *Pyrus communis *(pear); RAST: radio allergosorbent test; Que a: *Quercus alba *(oak); SPT: skin prick test; Syr v: *Syringa vulgaris *(lilac); Vig r: *Vigna radiata *(mungbean); Vit v: *Vitis vinifera *(grape); Zea m: *Zea mays *(maize)

## Competing interests

The authors declare that they have no competing interests.

## Authors' contributions

MH wrote the chapters on individual panallergen families. AR prepared figures and tables, and helped to classify known plant food allergens. FF conceived of the manuscript and participated in its design and discussion. ME wrote the introduction, participated in the design and discussion, and coordinated and drafted the manuscript. All authors read and approved the final manuscript.
